# AI‐assisted VMAT planning incorporating deep learning‐based dose prediction for head and neck cancer: feasibility of quality standardization and human intervention for irregular cases

**DOI:** 10.1002/acm2.70715

**Published:** 2026-07-26

**Authors:** Seiji Tomori, Kakutaro Narazaki, Shun Tasaka

**Affiliations:** ^1^ Department of Radiology National Hospital Organization Sendai Medical Center Sendai Miyagi Japan; ^2^ Department of Therapeutic Radiology, Course of Radiological Technology, Health Sciences Tohoku University Graduate School of Medicine Sendai Miyagi Japan

**Keywords:** deep learning, dose distribution prediction, head and neck cancer, quality standardization, VMAT

## Abstract

**Background:**

Volumetric‐modulated arc therapy (VMAT) represents a standard of care for head and neck cancer (HNC). However, treatment plan quality depends heavily on the planner's expertise, thereby challenging the standardization of treatment quality. While the clinical application of artificial intelligence (AI) in radiotherapy planning is rapidly increasing, the robustness of these models—for both typical and irregular cases—remains insufficiently understood.

**Purpose:**

To evaluate the clinical utility and generalizability of an artificial intelligence (AI)‐assisted volumetric‐modulated arc therapy (VMAT) planning workflow for head and neck cancer (HNC). This study focused on the robustness of AI‐assisted planning using dose distribution prediction for various cases including complex anatomical variations, such as bulky tumors and irregular tumor ruptures, and explored the feasibility of quality standardization through a “human‐in‐the‐loop” intervention approach via re‐optimization.

**Methods and Materials:**

Twelve VMAT plans from 11 HNC patients, including complex anatomical scenarios with bulky masses and post‐rupture anatomical changes, were analyzed. Clinical plans developed by an experienced planner were compared with AI‐assisted plans generated using RatoGuide (AiRato Inc.). The AI‐assisted planning workflow utilized deep learning‐based dose distribution prediction to generate dose‐derived ring contours for optimization. A human‐in‐the‐loop intervention was implemented, wherein planners performed manual fine‐tuning if initial AI‐assisted plans violated the institutional dose constraint of Dmax <115%. Dosimetric metrics for the planning target volume (PTV) and organs at risk (OARs), along with Paddick conformity index and monitor units (MU), were evaluated.

**Results:**

AI‐assisted plans achieved PTV coverage comparable to clinical plans while improving OAR sparing, particularly for the spinal cord and brainstem. The mean spinal cord PRV Dmax was reduced from 27.50 Gy in clinical plans to 23.97 Gy in AI‐assisted plans. Even in cases with bulky or ruptured tumors, AI‐assisted planning maintained high conformity and the overall mean Paddick conformity index was comparable to clinical plans. Although initial AI plans exhibited hotspots (Dmax > 115%) in three cases near the body surface, manual fine‐tuning of optimization successfully suppressed these hotspots, meeting clinical criteria. AI‐assisted planning maintained comparable mean MU values, and reduced the total planning time from approximately 2 h to 30 min.

**Conclusion:**

The AI‐assisted planning workflow demonstrated the potential to produce high‐quality VMAT plans, even for complex HNC cases. By integrating physicist human‐in‐the‐loop intervention with AI efficiency, this approach could facilitate the standardization of radiotherapy treatment planning quality while supporting patient safety.

## 1 INTRODUCTION

Intensity‐modulated radiation therapy (IMRT) and volumetric‐modulated arc therapy (VMAT) have become the standard of care for various malignancies, particularly head and neck cancer (HNC), due to their ability to deliver highly conformal dose distributions. These techniques improve planning target volume (PTV) coverage while simultaneously sparing adjacent organs at risk (OARs).^1^, ^2^, ^3^ IMRT and VMAT leverage complex multi‐leaf collimator (MLC) motion and dynamic dose rates to achieve these distributions; however, the planning process demands inverse planning optimization and final dose calculations within a treatment planning system (TPS).

To develop high‐quality plans tailored to individual patients, planners must iteratively refine optimization parameters in a trial‐and‐error process that is inherently time‐consuming, especially in complex anatomical cases like HNC. Furthermore, final plan quality depends heavily on the planner's expertise, which poses a challenge to the standardization of quality across different institutions and practitioners.^4^

To address these challenges, knowledge‐based planning (KBP) was introduced to improve efficiency and baseline quality by statistically utilizing prior clinical data.^5^, ^6^ KBP reduces variability in treatment plan quality by predicting achievable dose‐volume histograms (DVHs) based on anatomical structures, thereby contributing to increased planning efficiency and standardization.^7^, ^8^ Consequently, its practical implementation in commercial software has advanced significantly. Furthermore, the recent application of artificial intelligence (AI), particularly deep learning (DL), in radiation oncology has led to a surge in studies on dose distribution prediction and automated treatment planning.^9^, ^10^ Specifically, dose distribution prediction using DL‐based models, such as U‐Net, has demonstrated high utility across various anatomical sites.^11^, ^12^, ^13^, ^14^

Regarding AI‐assisted planning using predicted dose distributions, Kadoya et al. automated VMAT planning for prostate cancer,^15^ while Nemoto et al. evaluated its utility for lung stereotactic body radiation therapy (SBRT).^16^ Although AI‐driven VMAT planning has demonstrated clinical applicability across various treatment sites, HNC remains highly challenging due to its extremely complex target geometries and intertwined OARs.^17^ To address this, Saito et al. developed a dose distribution prediction model specifically for HNC patients treated with VMAT, achieving high predictive accuracy.^18^ Furthermore, Ishikawa et al. demonstrated the clinical feasibility of “RatoGuide” (AiRato Inc.), an AI‐assisted planning support tool utilizing deep learning‐based dose prediction, in a case study for HNC.^19^

However, the HNC region presents complex anatomical variations, and the robustness of AI models remains insufficiently understood for edge cases, such as those involving bulky tumors, tumor rupture, and subsequent structural alterations. To the best of our knowledge, few studies have specifically evaluated the clinical acceptability of AI‐assisted planning under these challenging conditions. In such edge cases, a fully automated workflow poses a risk of compromising patient safety; therefore, appropriate human intervention remains indispensable in a clinical setting.^20^ Further validation is warranted to assess the consistency and robustness of the AI‐assisted workflow across a broader clinical cohort, encompassing not only typical cases but also geometrically challenging edge cases.

The purpose of this study was to evaluate the clinical utility of a deep learning‐based workflow utilizing RatoGuide to generate dose‐derived contours for TPS optimization, specifically focusing on a diverse cohort of HNC cases, including bulky or ruptured tumors. Furthermore, we aimed to demonstrate a human‐in‐the‐loop approach that incorporates manual fine‐tuning to manage potential AI‐driven hotspots, thereby ensuring both planning efficiency and patient safety in clinical practice.

## 2 MATERIALS AND METHODS

### 2.1 Ethical approval

This study was approved by the Institutional Review Board of Sendai Medical Center (Approval No. 25‐61).

### 2.2 Patient characteristics and treatment methods

**FIGURE 1:**
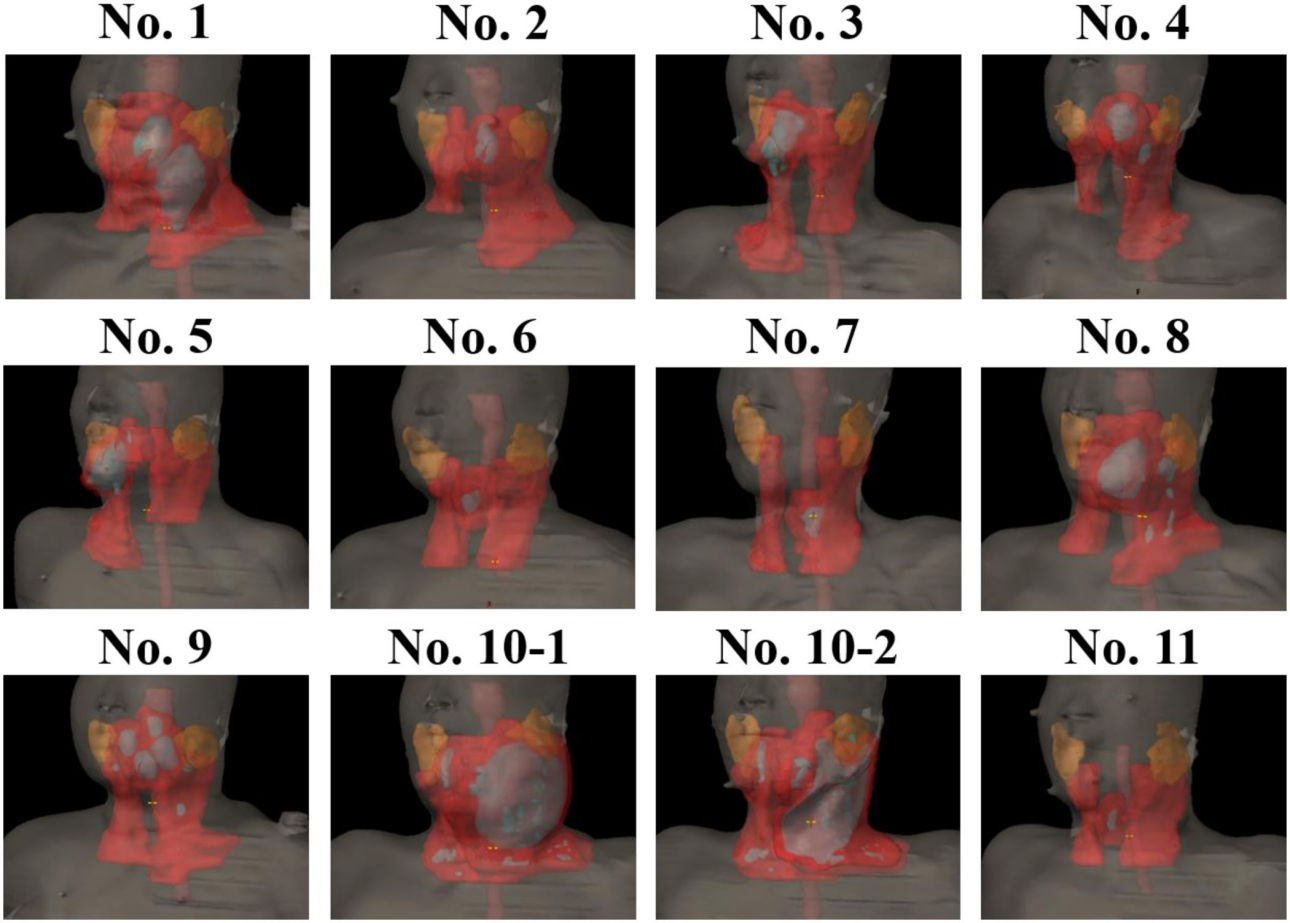
Three‐dimensional (3D) visualizations of the 12 cases from 11 patients included in this study. Cases No. 10‐1 and No. 10‐2 represent the same patient, illustrating a bulky tumor mass (10‐1) and the subsequent anatomical changes following tumor rupture (10‐2). Contours are color‐coded as follows: GTV (cyan), PTV (red), planning organ at risk volumes (PRVs) for the spinal cord and brainstem (pink), and bilateral parotid glands (orange). The brainstem was not delineated in Case No. 11 as it was located outside the treatment field.

A total of 12 VMAT plans from 11 patients with HNC treated at our institution were retrospectively analyzed. Figure 1 illustrates the external views of representative cases. The HNC radiotherapy protocol consisted of a sequential boost technique: an initial phase targeting the prophylactic nodal stations, followed by a localized boost to the tumor. This study evaluated the initial‐phase treatment plans, which are characterized by inherently complex target volumes and dose distributions. Cases No. 10‐1 and 10‐2 shown in Figure 1 represent the same patient: No. 10‐1 is the initial plan for a bulky tumor, and No. 10‐2 is the replan generated following tumor rupture. The cohort consisted of eight male and three female patients, with ages ranging from 51 to 75 years (median: 69 years). The targets and OARs were manually delineated by two radiation oncologists according to the 2013 international consensus guidelines for cervical lymph node levels.^21^ The specific oncologist responsible for each plan is detailed in Table S1 (Supplementary Material). The PTV margin applied in this study was 5 mm. All treatments were delivered using a TrueBeam STx linear accelerator (Varian Medical Systems, Palo Alto, CA, USA) with 6 MV X‐rays. The TPS utilized was Eclipse (version 15.1, Varian Medical Systems, Palo Alto, CA, USA). CT images were acquired with a 2 mm slice thickness, and the dose calculation grid size was set to 2 mm. The Acuros XB algorithm was utilized for the final dose calculation.

### 2.3 Clinical planning process

Clinical plans used in actual treatments were developed according to our institutional dose constraints. The constraints for the PTV (excluding air) were D2% < 110% and Dmax < 115%. For OARs, the following criteria were applied: spinal cord PRV Dmax < 45 Gy, brainstem PRV Dmax < 54 Gy, parotid gland Dmean < 26 Gy (for at least one side). These constraints were based on a total dose of 70 Gy delivered in two phases: an initial phase (including prophylactic lymph node regions) and localized tumor boost. This study focused specifically on the initial‐phase plans, which exhibit greater complexity in dose distribution. During the initial phase, OAR doses were minimized as much as possible to accommodate the subsequent boost dose. In cases where parotid glands overlapped with the PTV, planners prioritized dose reduction to the contralateral parotid gland to ensure that at least one side met the criterion. In this retrospective study, the prescription dose for the initial irradiation plans was defined as 40 Gy in 20 fractions. To compare the conformity of dose distributions, normalization was set to the PTV D50% (i.e., D50% = 40 Gy).^22^ These prescription dose and normalization settings were applied consistently to both clinical and AI‐assisted plans detailed below. Regarding the arc configurations, nine clinical plans utilized two full arcs, while the remaining three utilized two full arcs and one‐half arc. The specific gantry and collimator angles for each plan are summarized in Table S2 (Supplementary Material). The planning time for the clinical plans was approximately 2 h per case.

### 2.4 AI‐assisted planning process

For AI‐assisted planning, we utilized RatoGuide (version 1.6.1.6; AiRato Inc., Sendai, Japan), a deep learning‐based prototype software designed for treatment planning. The RatoGuide model used in this study was developed based on the dense dilated U‐Net architecture for three‐dimensional (3D) dose prediction, as proposed by Gronberg et al.^23^ It predicts dose distributions by utilizing CT images and DICOM‐RT structure sets (i.e., target volumes and OARs) as input data.

In the RatoGuide prototype employed in this study, users can choose between two prediction models: one trained on plans optimized with a priority on PTV dose coverage, and another trained on plans aimed at further reducing OAR doses while maintaining PTV coverage. This choice specifically influences dose distribution within PTV‐OAR overlap regions. In accordance with our institution's treatment planning policy, we selected the latter model to prioritize OAR sparing.

**FIGURE 2:**
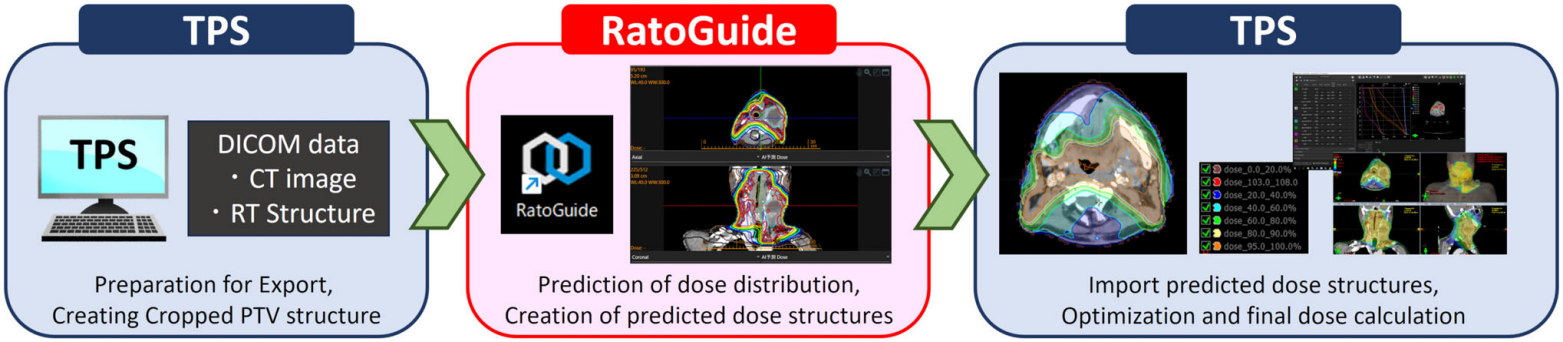
Schematic workflow of the AI‐assisted planning process using RatoGuide in this study.

Once the dose distribution prediction is generated, seven dose‐derived structures corresponding to dose ranges of 0%‐20%, 20%‐40%, 40%‐60%, 60%‐80%, 80%‐90%, 95%‐100%, and 103%‐108% of the prescription dose are automatically extracted from the predicted distribution. These structures are created to serve as optimization objectives within the TPS.^15^, ^16^ Because these generated structures can be imported into any standard TPS for the final optimization process, the RatoGuide‐integrated workflow proposed in this study is inherently adaptable to various commercial planning platforms. The specific optimization workflow is illustrated in Figure 2 and proceeds as follows:

1. TPS pre‐processing and data export: A “cropped PTV” was manually generated by contracting the original PTV by 3 mm from the body surface. This pre‐processing step accounts for the inherent limitations of the TPS in accurately modeling doses within the skin buildup region. Under conditions of electronic disequilibrium, enforcing prescription doses within this region can inadvertently cause severe over‐optimization, resulting in localized hotspots beyond the depth of maximum dose. After pre‐processing, DICOM data, including CT images and RT structure sets, were manually exported from the TPS and imported into RatoGuide software.
2. Dose distribution prediction and structure generation: In the RatoGuide software, the deep learning model automatically predicted the dose distribution based on the patient's anatomy and structure sets. Subsequently, seven dose‐derived structures (corresponding to dose ranges of 0%‐20%, 20%‐40%, 40%‐60%, 60%‐80%, 80%‐90%, 95%‐100%, and 103%‐108% of the prescription dose) were automatically generated from the predicted distribution. These structures were then manually imported back into the TPS as DICOM RT‐structure.
3. Optimization and human‐in‐the‐loop intervention: Optimization objectives were manually set for the seven AI‐generated structures and the cropped PTV using the developer‐recommended values (Table 1, left). If the Dmax of the Body structure exceeded 115% of the prescription dose (our institutional safety threshold), these regions were identified as unacceptable hotspots. To address this, manual fine‐tuning was integrated as a human‐in‐the‐loop intervention, whereby the optimization constraints were adjusted (Table 1, right) prior to initiating re‐optimization. All AI‐assisted plans were configured with a standardized geometry of two full arcs with consistent collimator angles, as recommended by the developer (detailed in Table S2, Supplementary Material).

**TABLE 1:** Optimization parameters for AI‐assisted planning.

Recommended optimization dose constraints					Manual fine‐tuning dose constraints				
	Type	Volume [%]	Dose [cGy]	Priority		Type	Volume [%]	Dose [cGy]	Priority
PTV	lower	100	3900	850	PTV	lower	99.9	3900	850
	lower	99.9	4050	800		lower	99.5	4050	800
	upper	0	4010	600		upper	0	4010	600
						upper	0	4200	850
Dose ring					Dose ring				
0%‐20%	upper	0	800	800	0‐20%	upper	0	800	800
20%‐40%	upper	0	1400	800	20‐40%	upper	0	1400	800
40%‐60%	upper	0	2200	800	40‐60%	upper	0	2200	800
60‐80%	upper	0	3100	650	60‐80%	upper	0	3100	650
80%‐90%	upper	0	3600	550	80‐90%	upper	0	3600	550
95%‐100%	upper	0	4010	600	95‐100%	upper	0	4010	600
103%‐108%	upper	0	4120	500	103‐108%	upper	0	4120	500

### 2.5 Objects for dosimetric comparison and plan evaluation

We compared the clinical plans used in actual treatments with the AI‐assisted plans using specific dose‐volume histogram (DVH) metrics. Target coverage was evaluated using D2%, mean dose (Dmean), D98%, and maximum dose (Dmax) for the PTV (excluding air), and D2%, Dmean, and D98% for the CTV. For OARs, Dmax and Dmean were assessed for the spinal cord and brainstem PRVs (defined with a 2‐mm margin), alongside the Dmean for the ipsilateral and contralateral parotid glands, and Dmax for the Body structure.

Furthermore, the Paddick Conformity Index (CI) was employed to evaluate dose conformity.^24^ Because dose normalization was performed at the PTV D50%, the maximum achievable Paddick CI value is approximately 0.5 due to the mathematical definition. To ensure that the AI‐assisted plans did not exhibit excessive delivery complexity, the total number of Monitor Units (MUs) was also compared as a metric for delivery efficiency.

Statistical comparisons between clinical and AI‐assisted plans were performed using the Wilcoxon signed‐rank test to account for the small sample size (*n* = 12). A *p*‐value of < 0.05 was considered statistically significant. Statistical analyses were performed using EZR (Saitama Medical Center, Jichi Medical University, Saitama, Japan),^25^ which is a graphical user interface for R (The R Foundation for Statistical Computing, Vienna, Austria).

## 3 RESULTS

### 3.1 Dosimetric comparison

The overall mean values and standard deviations for the PTV parameters across the 12 plans for the clinical vs. AI‐assisted planning were as follows: D98% (34.47 ± 2.42 vs. 34.00 ± 2.25 Gy, *p*<0.01), Dmean (39.65 ± 0.16 vs. 39.67 ± 0.14 Gy, *p *= 0.16), D2% (41.69 ± 0.40 vs. 42.23 ± 0.75 Gy, *p*<0.01), and Dmax (43.72 ± 0.73 vs. 45.53 ± 1.97 Gy, *p*<0.01). For the CTV, the corresponding values were: D98% (37.51 ± 2.04 vs. 38.05 ± 1.93 Gy, *p*<0.01), Dmean (40.22 ± 0.12 vs. 40.37 ± 0.13 Gy, *p*<0.01), and D2% (41.82 ± 0.42 vs. 42.43 ± 0.88 Gy, *p*<0.01). Evaluation of the Paddick CI for the PTV yielded 0.479 ± 0.007 for the clinical plans and 0.478 ± 0.009 for the AI‐assisted plans (*p *= 1.00), demonstrating no significant difference between the two planning methods.

Regarding the OARs, the AI‐assisted plans significantly reduced doses to the spinal cord PRV, with lower values observed for both Dmax (27.50 ± 1.65 vs. 23.97 ± 2.12 Gy, *p*<0.01), and Dmean (17.69 ± 2.03 vs. 12.92 ± 1.65 Gy, *p*<0.01). Similarly, for the brainstem PRV, the AI‐assisted plans achieved reductions in both Dmax (23.97 ± 4.07 vs. 21.59 ± 5.47 Gy, *p *= 0.03) and Dmean (7.73 ± 3.50 vs. 5.49 ± 2.60 Gy, *p*<0.01). Parotid gland sparing was inconsistent: the clinical plans demonstrated superiority for the contralateral parotid gland Dmean (16.82 ± 2.30 vs. 20.16 ± 4.70 Gy, *p*<0.01), whereas the AI‐assisted plans were superior for the ipsilateral parotid gland Dmean (21.44 ± 5.55 vs. 19.28 ± 6.74 Gy, *p *= 0.01).

The mean Body Dmax was 43.78 ± 0.71 Gy for the clinical plans and 45.53 ± 1.97 Gy for the initial AI‐assisted plans (*p *= 0.01). However, in three AI‐assisted plans (Nos. 3, 4, and 10‐2), the Body Dmax exceeded 115% of the prescription dose, reaching 119.94%, 121.00%, and 123.65%, respectively.

### 3.2 Overall dose distribution and DVH

**FIGURE 3:**
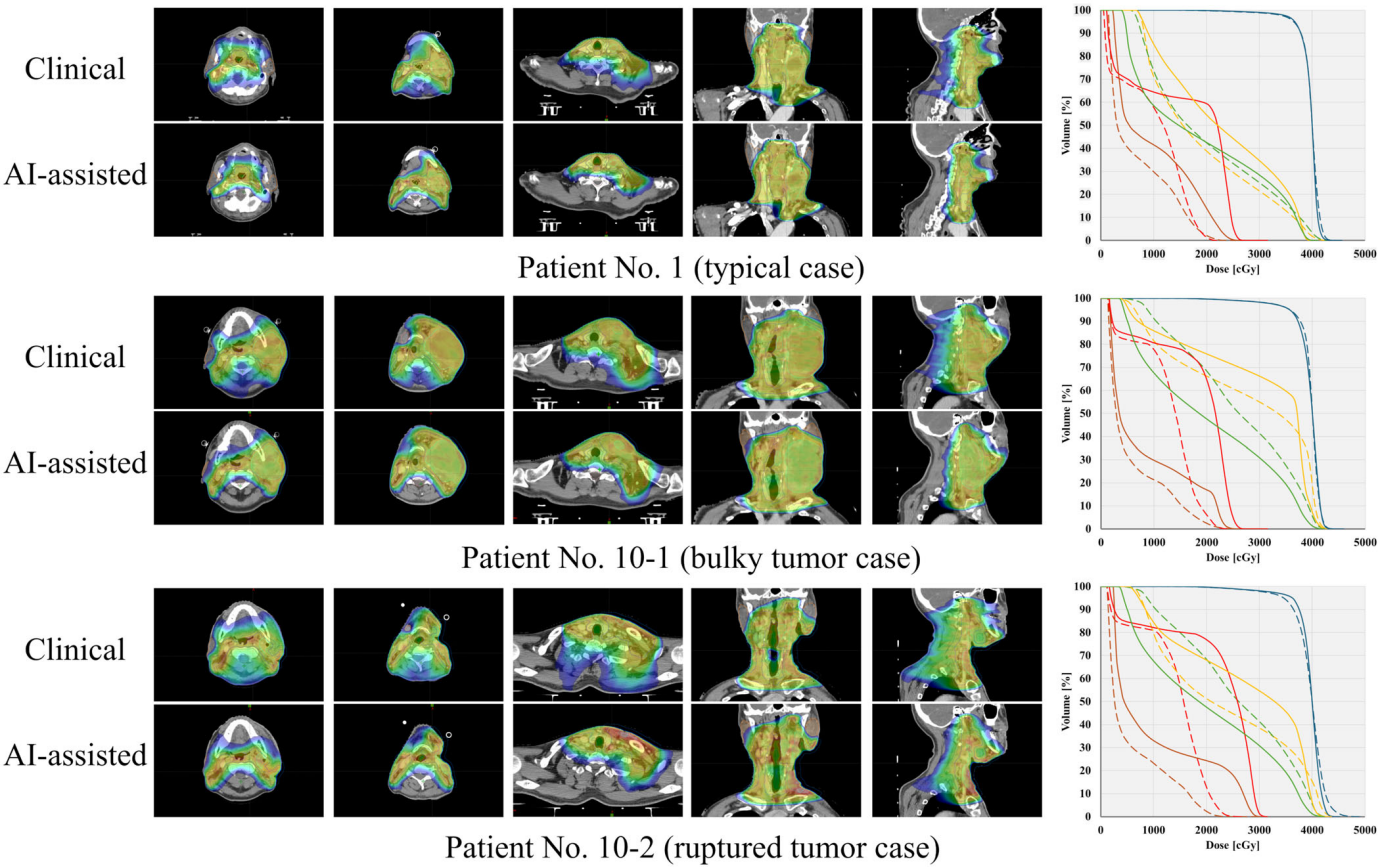
Plan comparison between the clinical and AI‐assisted approaches for three representative cases (a typical tumor, a bulky tumor, and an irregularly shaped ruptured tumor). Note: The color scale for the dose distributions ranges from 50% to 115% of the prescription dose. Regions exceeding 115% are highlighted in pink within the color wash to represent areas above the upper safety threshold (observed in specific areas near the skin surface of No. 10‐2). In the dose‐volume histograms (DVHs), the solid and dashed lines represent the clinical plans and AI‐assisted plans, respectively. The color‐coded curves represent the following structures: blue, PTV; red, spinal cord PRV; brown, brainstem PRV; green, contralateral parotid gland; and yellow, ipsilateral parotid gland.

**FIGURE 4:**
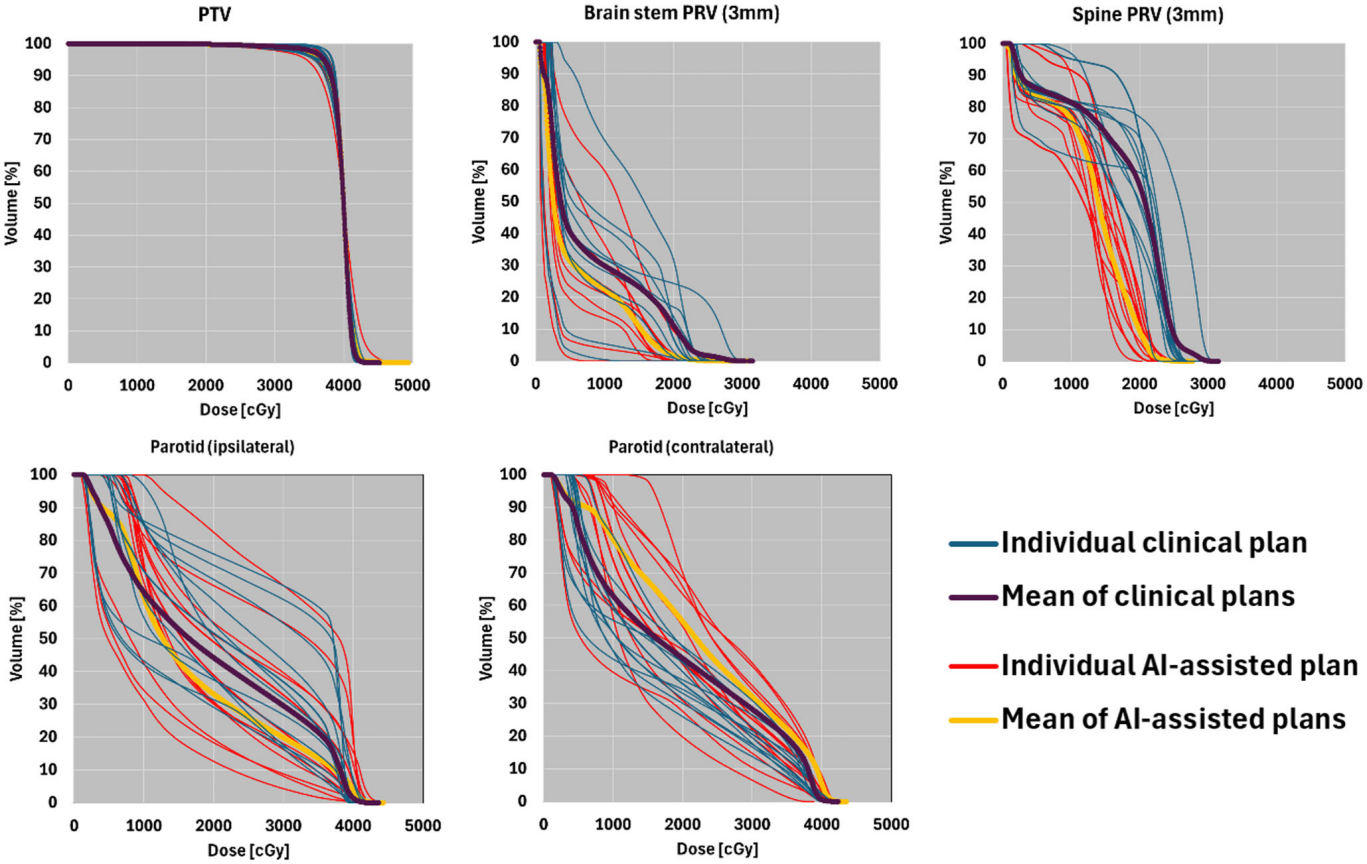
Population dose‐volume histograms (DVHs) across all 12 cases, comparing individual datasets and overall averages between the clinical and AI‐assisted approaches. Thin blue and red lines represent the individual clinical and AI‐assisted plans, respectively. Thick purple and yellow lines represent the mean DVHs for the clinical and AI‐assisted plans averaged over all 12 cases.

Figure 3 displays representative dose distributions and DVH comparisons for three distinct scenarios: a typical case (No. 1), a case with a bulky tumor (No. 10‐1), and a case with a ruptured tumor (No. 10‐2). The AI‐assisted plans demonstrated a superior reduction in the mid‐to‐low dose regions surrounding the PTV. Regarding the PTV, the AI‐assisted plans provided target coverage comparable to that of the clinical plans. However, consistent with the individual metric analysis, the AI‐assisted plans achieved enhanced sparing of the ipsilateral parotid gland, whereas the clinical plans excelled in sparing the contralateral parotid gland. The population DVH for all 12 cases is shown in Figure 4.

### 3.3 Hotspot reduction via manual fine‐tuning

**FIGURE 5:**
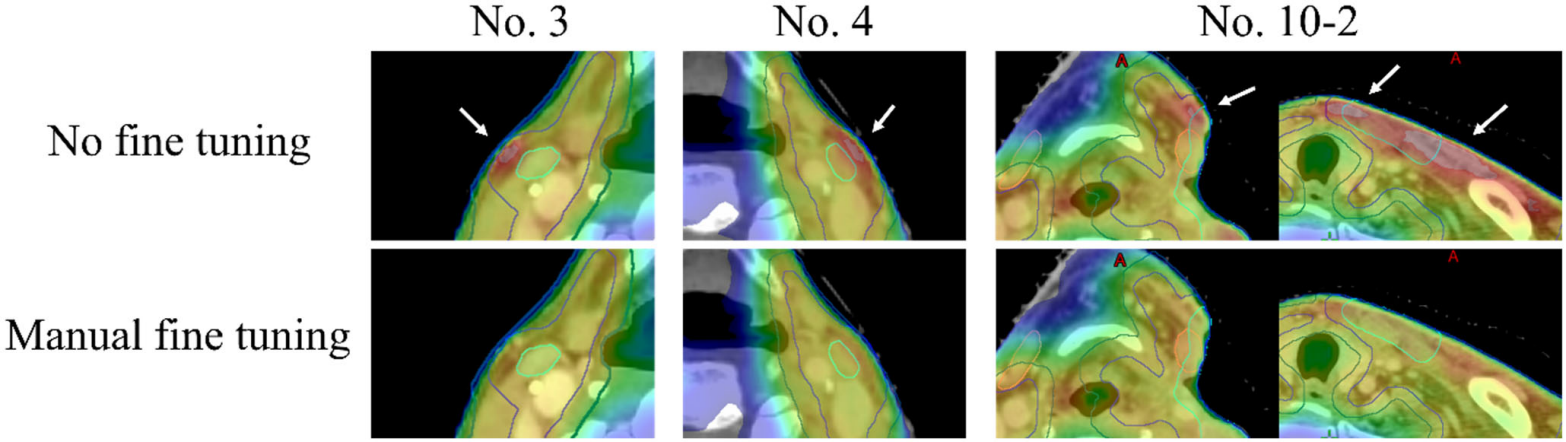
Reduction of hotspots (>115% of the prescription dose) in three initial AI‐assisted plans via manual fine‐tuning. The color scale for dose distribution ranges from 50% to 115% of the prescription dose. Regions exceeding 115% are highlighted in pink within the color wash to represent areas above the upper threshold.

Regarding the three initial AI‐assisted plans that contained hotspots (No. 3, 4, and 10‐2, as described in Section 3.1), manual fine‐tuning was applied and successfully resolved these hotspots, reducing the Dmax values to 109.68%, 109.55%, and 111.03%, respectively, thereby meeting our institutional criteria. Figure 5 illustrates the changes in the dose distribution before and after manual fine‐tuning. The overall mean Paddick CI across the 12 AI‐assisted plans, including the three fine‐tuned cases, was 0.483 ± 0.007, which showed no substantial difference compared with the clinical plans (*p *= 0.81).

### 3.4 Monitor units

The overall mean MU values were 654.9 ± 58.1 for the clinical plans and 632.9 ± 52.7 for the initial AI‐assisted plans (*p *= 0.34). When including the three cases that underwent manual fine‐tuning, the mean MU value for the AI‐assisted plans was 628.8 ± 44.2 (*p *= 0.23). There was no statistically significant difference between the clinical and AI‐assisted plans, demonstrating that the delivery efficiency remained highly comparable.

## 4 DISCUSSION

In this study, we evaluated the RatoGuide‐based AI‐assisted planning system using 12 plans across 11 HNC patients. Our results demonstrated that all AI‐assisted plans maintained sufficient target coverage for the PTV while effectively reducing doses to the OARs, particularly the brainstem and spinal cord. Regarding the parotid glands, the dose to the ipsilateral parotid gland was lower in the AI‐assisted plans, whereas the dose to the contralateral parotid gland was higher than that in the clinical plans. This discrepancy likely stems from our institutional policy, which prioritizes sparing the contralateral parotid gland over strict PTV coverage to ensure that at least one parotid gland meets the requisite dose constraints. However, the lower dose achieved by the AI‐assisted plans for the ipsilateral parotid gland suggests a potential for further dose reduction beyond what is achievable with manual clinical planning. The Paddick CI showed no significant difference between the clinical and AI‐assisted plans, indicating that the RatoGuide‐integrated workflow ensures a level of dose conformity to the PTV equivalent to that of manual clinical planning.

Importantly, even in clinically challenging scenarios involving bulky tumors or highly irregular target geometries caused by tumor rupture (e.g., No. 10‐1 and 10‐2, as shown in Figure 3), the RatoGuide‐based AI‐assisted workflow generated plans with superior suppression of the mid‐to‐low dose regions and sufficient dose conformity compared with the manual clinical plans. This suggests that our AI‐assisted planning workflow, when integrated with human‐in‐the‐loop intervention, is sufficiently robust for clinical implementation, even in such complex edge‐case scenarios. Compared with previous studies that focused primarily on the average dose distribution prediction accuracy within standard patient cohorts, our study highlights the robust performance and clinical adaptability of this workflow when confronting geometrically challenging anatomy.^18^, ^19^

Conversely, the initially calculated AI‐assisted plans using the vendor‐recommended optimization parameters exhibited a tendency toward an elevated maximum dose near the body surface, exceeding our institution's tolerance in three cases. This discrepancy is likely because the optimization parameters recommended by the vendor are tuned to deliver the prescription dose to nearly 100% of the PTV volume; this includes regions near the skin surface to compensate for the inherent dose fall‐off within the electronic build‐up region. To address this, we manually adjusted the optimization parameters based on our clinical experience, successfully suppressing Dmax to below 115% of the prescription dose. This confirms that the combination of AI‐predicted dose distributions and the resultant dose‐derived structures can yield high‐quality clinical plans within a short timeframe (approximately 30‐60 min) when appropriate planning parameters are manually adjusted by the user.

A key finding of this study is that the AI‐assisted planning workflow provides consistent, high‐quality plans regardless of the planner's individual experience or technical proficiency. As shown in the DVH results (Figures 3 and 4), the AI‐assisted plans reduced doses to the spinal cord and brainstem compared with the clinical plans in many cases. The higher OAR doses observed in the clinical plans may be attributed to the fact that human planners must prioritize fulfilling stringent mandatory constraints for multiple organs within limited working hours; this often leaves insufficient time to further minimize and optimize the mid‐to‐low dose regions.

Although the clinical plans met the requisite institutional constraints, OAR doses should be minimized to the lowest achievable levels, especially considering the potential necessity for re‐irradiation in the event of local recurrence. By utilizing this workflow based on predicted dose distributions, AI‐assisted planning enables the creation of consistently optimized plans that are free from the constraints of clinical time limits or intra‐planner variability. The implementation of this approach is anticipated to significantly contribute to the standardization and quality improvement of radiotherapy planning while simultaneously enhancing clinical workflow efficiency.

Furthermore, while knowledge‐based planning (KBP) methods based on anatomical information are increasingly being integrated into modern treatment planning systems, this AI‐assisted planning workflow does not compete with KBP but rather offers the potential to synergistically leverage the strengths of both approaches. KBP can predict dose‐volume histograms (DVHs) that reflect institutional preferences by learning from past clinical plans.^7^, ^8^ However, since KBP is inherently dependent on the quality of the training dataset, it carries the risk of leading to suboptimal outcomes if the underlying data are insufficient or inconsistent. By integrating our method, it becomes possible to maintain a consistently high standard of quality while utilizing KBP to reflect institutional policies and achieve superior optimization tailored to individual patients. Although this potential synergy warrants further investigation, subsequent studies are anticipated to validate these combined benefits.

The MU values for the AI‐assisted plans demonstrated no significant difference compared with those of the clinical plans. This suggests that the AI‐assisted planning yields high‐quality plans without introducing overly complex MLC modulation, thereby ensuring high deliverability during clinical treatment delivery. Furthermore, the planning time for the AI‐assisted planning in this study was approximately 30 min, which is consistent with the findings previously reported by Ishikawa et al.^19^ Even if an initial AI‐assisted plan fails to fully satisfy clinical requirements, a revised plan can be finalized within an additional 15‐30 min, inclusive of manual fine‐tuning, re‐optimization, and final dose calculation.

Since this workflow using RatoGuide is not fully autonomous, there are inherent limits to further reducing the total planning time at this stage. However, the primary advantage of this approach is not merely time reduction, but rather the “margin for intervention” it affords. This allows planners to verify anatomical structures and actively intervene in both the optimization and final dose calculation processes within the TPS. While standardization is highly desirable, clinicians frequently confront a dilemma where treatment plans must be customized based on complex, patient‐specific anatomy and clinical conditions. Fully autonomous systems raise concerns regarding the management of atypical cases and the potential atrophy of critical clinical judgment skills among practitioners. Responsibility for any unintentional discrepancies and the final plan quality ultimately resides with the human professional under existing legal and clinical frameworks; consequently, the human‐in‐the‐loop workflow implemented in this study aligns with the recommendations of current guidelines.^20^, ^26^, ^27^

At the current stage of AI development, we believe that a “human‐in‐the‐loop” approach—where human experts perform final adjustments based on AI‐predicted insights—is the most appropriate for clinical integration. This approach does not simply use AI to replace the planner; rather, it uses the AI as a high‐level “guidance system for achievable quality” that the medical physicist validates against the physical laws of radiation therapy. Responsibility for final plan quality remains with the human professional, but AI‐assisted planning provides the efficient tools to achieve a higher standard of care more consistently. Medical physicists, who possess comprehensive knowledge of the real physical environment ‐such as linear accelerator commissioning, selection and validation of calculation algorithms, patient‐specific quality assurance (QA)‐ should ultimately judge plan acceptability.

While previous literature has demonstrated the feasibility of RatoGuide in individual cases, this study is, to the best of our knowledge, the first to comprehensively evaluate the optimization process using dose‐level structures in a cohort with significant anatomical variations and irregular cases. A key contribution of this work is the incorporation of a specific strategy for human intervention to resolve unintended dose distributions (e.g., hotspots) that may arise during the AI‐assisted process.

Unlike “black‐box” fully automated systems, our workflow allows experts to maintain control over the optimization process via the AI‐generated structures. This provides high interpretability and ensures safety through expert oversight. By balancing the benefits of AI (efficiency and standardization) with professional judgment (safety and handling of complex cases), this study demonstrates a feasible model for a new clinical standard in the era of AI‐integrated radiotherapy.

Finally, we acknowledge several limitations of this study. This research was conducted at a single institution with a relatively small sample size of 12 cases. Therefore, further clinical validation with a larger cohort is required to confirm these preliminary results. By addressing these limitations in future studies, we believe this method has the potential to contribute to the standardization of plan quality while maintaining rigorous expert oversight, thereby representing a highly feasible workflow model for balanced semi‐automation.

The importance of the experience and knowledge of medical physicists remains fundamental in this workflow. However, to safely and effectively integrate advanced AI technologies into clinical practice, there is an urgent need to develop comprehensive educational frameworks and clear institutional guidelines addressing proper AI utilization, technological limitations, and professional accountability. Ultimately, these findings are anticipated to significantly contribute to the standardization of VMAT planning, thereby facilitating the realization of safer and more consistent radiation therapy.

## 5 CONCLUSION

This study suggests that AI‐assisted planning using RatoGuide has the potential to achieve high‐quality VMAT plans with favorable OAR sparing compared with manual clinical plans for HNC, even in individual cases involving bulky or irregularly shaped tumors. By facilitating standardized plan generation, this approach appears to offer promising clinical utility within the scope of this preliminary evaluation. Furthermore, this methodology appears to align with contemporary guidelines for clinical AI integration, which seek to ensure safety and expert oversight while balancing plan quality and workflow efficiency. Consequently, this pragmatic human‐in‐the‐loop approach may help mitigate the uncertainties associated with full automation, potentially supporting safe, individualized treatment under expert supervision.

## AUTHOR CONTRIBUTIONS

**Seiji Tomori**: Conceptualization; data curation; formal analysis; investigation; methodology; project administration; supervision; resources; validation; visualization; writing—original draft preparation; writing—review & editing. **Kakutaro Narazaki**: Data curation; investigation; methodology; resources; supervision; writing—review & editing. **Shun Tasaka**: Data curation; investigation; methodology; resources; supervision; writing—review & editing.

## CONFLICT OF INTEREST STATEMENT

The authors received grants from AiRato Inc.

## Supporting information




**Supporting file 1**: acm270715‐sup‐0001‐TableS1.docx

**Supporting file 2**: acm270715‐sup‐0002‐TableS2.docx
