# Supplementary material for: AI‐assisted VMAT planning incorporating deep learning‐based dose prediction for head and neck cancer: feasibility of quality standardization and human intervention for irregular cases
Source: J Appl Clin Med Phys. 2026 Jul 26;27(8):e70715. doi: 10.1002/acm2.70715 (PMC13402453; doi:10.1002/acm2.70715)
Supplement: Supplementary file 1 — Supporting file 1: acm270715‐sup‐0001‐TableS1.docx [file ACM2-27-e70715-s001.docx]

Table S1 Radiation oncologists in charge of contouring for each plan

| Plan No. | No. 1 | No. 2 | No. 3 | No. 4 | No. 5 | No. 6 | No. 7 | No. 8 | No. 9 | No. 10-1 | No. 10-2 | No. 11 |
| --- | --- | --- | --- | --- | --- | --- | --- | --- | --- | --- | --- | --- |
| Oncologist No. | 1 | 2 | 2 | 2 | 2 | 2 | 2 | 1 | 1 | 1 | 1 | 2 |
| * "1" and "2" denote the specific radiation oncologists who performed the contouring for each case. | | | | | | | | | | | |  |
