# Supplementary material for: AI‐assisted VMAT planning incorporating deep learning‐based dose prediction for head and neck cancer: feasibility of quality standardization and human intervention for irregular cases
Source: J Appl Clin Med Phys. 2026 Jul 26;27(8):e70715. doi: 10.1002/acm2.70715 (PMC13402453; doi:10.1002/acm2.70715)
Supplement: Supplementary file 2 — Supporting file 2: acm270715‐sup‐0002‐TableS2.docx [file ACM2-27-e70715-s002.docx]

Table S2 Arc configuration for each plan

| Clinical plan | |  |  |  |  |  |  |  |  |  |  |  |  |
| --- | --- | --- | --- | --- | --- | --- | --- | --- | --- | --- | --- | --- | --- |
| Plan No. | | No. 1 | No. 2 | No. 3 | No. 4 | No. 5 | No. 6 | No. 7 | No. 8 | No. 9 | No. 10-1 | No. 10-2 | No. 11 |
| Arc 1 | Gantry rotation | 181 -179 | 181 -179 | 181 -179 | 181 -179 | 181 -179 | 181 -179 | 181 -179 | 181 -179 | 181 -179 | 179 - 181 | 181 -179 | 181 -179 |
|  | Collimator angle | 30 | 25 | 30 | 30 | 20 | 20 | 30 | 25 | 335 | 340 | 20 | 25 |
| Arc 2 | Gantry rotation | 179 - 181 | 179 - 181 | 179 - 181 | 179 - 181 | 179 - 181 | 179 - 181 | 179 - 181 | 179 - 181 | 179 - 181 | 181 -179 | 179 - 181 | 179 - 181 |
|  | Collimator angle | 330 | 335 | 330 | 330 | 340 | 340 | 330 | 335 | 25 | 20 | 340 | 335 |
| Arc 3 | Gantry rotation |  |  |  |  |  |  |  | 181 - 0 | 179 - 0 | 179 - 0 |  |  |
|  | Collimator angle |  |  |  |  |  |  |  | 25 | 335 | 340 |  |  |
|  |  |  |  |  |  |  |  |  |  |  |  |  |  |
| AI-assisted plan | |  |  |  |  |  |  |  |  |  |  |  |  |
| Plan No. | | All plans | | | | | | | | | | | |
| Arc 1 | Gantry rotation | 181 - 179 | | | | | | | | | | | |
|  | Collimator angle | 23 | | | | | | | | | | | |
| Arc 2 | Gantry rotation | 179 - 181 | | | | | | | | | | | |
|  | Collimator angle | 293 | | | | | | | | | | | |
| * All arc arrangements of AI-assisted plan were set to values recommended by the developer of RatoGuide. | | | | | | | | | | | | | |
